# Validation of the anxiety subscale of the Spanish version of the Edinburgh Postnatal Depression Scale (EPDS-A)

**DOI:** 10.3389/fpsyt.2023.1294206

**Published:** 2023-12-11

**Authors:** Marta Gomà, Leire Gordo, Eric Cozodoy, Leire Iriarte, Emma Prims, Josep Ferrer, Carmen Sánchez, Antònia Llairó, Eulàlia Arias-Pujol

**Affiliations:** ^1^Bruc Salut, Barcelona, Spain; ^2^Deusto FamilyPsych, Facultad de Ciencias de la Salud, Universidad de Deusto, Bilbao, Spain; ^3^Facultat de Psicologia, Ciències de l’Educació i de l’Esport Blanquerna, Universitat Ramon Llull, Barcelona, Spain; ^4^CAP Roquetes-Canteres, Institut Català de la Salut, Barcelona, Spain

**Keywords:** EPDS-A, anxiety, detection, perinatal, screening, prevention

## Abstract

**Introduction:**

There is a need to implement routine perinatal mental health screening in Spain. Therefore, it is necessary to systematise the detection of depressive and anxious symptoms in pregnancy and postpartum using the same instrument. The Edinburgh Postnatal Stress Depression Scale (EPDS) is frequently used as a rapid, effective and cross-culturally validated screening tool for perinatal depression. In several countries, an Anxiety subscale, the EPDS-A, was identified within the EPDS. Although the factorial structure of the EPDS has been investigated in Spanish population, the EPDS-A has not yet been validated. This study aimed to validate the EPDS-A as a measure of perinatal anxiety in Spanish population.

**Methods:**

161 women were evaluated with the EPDS and the State–Trait Anxiety Inventory (STAI) during pregnancy and postpartum. Confirmatory factor analysis (CFA) was used to confirm the trifactorial structure of the EPDS, comprising the dimensions of Depression, Anhedonia and Anxiety. Likewise, the invariance of the trifactorial model between pregnancy and postpartum was tested. Finally, the correlations between the EPDS-A and the STAI subscales (State Anxiety and Trait Anxiety) were calculated.

**Results:**

The Exploratory factor analysis (EFA) driven three-factor structure of the EPDS, consisting of an Anhedonia factor (Items 1, 2, and 10), an Anxiety factor (Items 3, 4, 5, and 6) and a Depression factor (Items 7, 8, and 9), was the best measurement model for the current data compared to the alternative model tested [χ^2^ = 34.592, *df* = 32, *p* = 0.34; χ^2^/*df* = 1.08; RMSEA = 0.023, 90% Confidence Interval [CI] [0.000, 0.064], CFI = 0.996, GFI = 0.960]. The model’s invariance between pregnant and postpartum women was confirmed. The existence of an Anxiety subscale within the EPDS was also confirmed. The scores obtained with the EPDS-A correlated moderately with scores on both subscales of the STAI during pregnancy and after delivery. Using the STAI as a criterion and prioritising the instrument’s sensitivity, a cut-off point of 4 points was established for the EPDS-A.

**Conclusion:**

Our results confirm the trifactorial structure of the EPDS in Spanish population. The Anxiety subscale was validated for routine perinatal mental health screening.

## 1 Introduction

Despite the high prevalence (1), perinatal anxiety is usually underestimated during pregnancy (2), and less diagnosed than depression (3).

In a recent meta-analysis, it has been determined that approximately 20.7% of women display traits associated with anxiety disorders, with a notable tendency towards higher prevalence during pregnancy in contrast to the postpartum phase (4). The study’s findings indicate that 5.5% of women meet the diagnostic criteria for a minimum of two distinct anxiety disorders, with specific phobia, generalised anxiety disorder, and social phobia emerging as the most frequently observed manifestations of perinatal anxiety.

Recently, researchers have questioned the need to screen for both symptoms (5), finding higher rates since covid (depression symptoms 26.7% in pregnancy and 32.7% in postpartum; anxiety symptoms 20% in pregnancy and 26.6% in postpartum; comorbidity was found to be 15.2% in pregnancy and 20.3% in postpartum) (6). Perinatal anxiety is associated with a higher severity of symptoms and has a negative impact on both mothers and the development of their offspring (7–9). About 80% of women are not diagnosed with anxiety and/or depression and do not receive adequate treatment, or the diagnosis is late when these conditions are already severe and generalised (10). Therefore, timely identification of women at risk would allow implementing preventive interventions during pregnancy to reduce the likelihood that these women will experience full episodes of depression and anxiety (10).

Antenatal anxiety correlates with a high likelihood of experiencing preterm birth, spontaneous preterm birth, reduced mean birth weight, an increased likelihood of low birth weight, a shorter gestational age, the baby’s elevated risk of being small for gestational age, and a smaller head circumference (11). Failing to address anxiety disorders heightens the susceptibility to postpartum depression and is linked to diminished maternal self-assurance, early complications in the offspring (such as behavioural inhibition, difficulties in mother-infant interaction, and insecure attachment), as well as subsequent adverse child development (12). The presence of maternal anxiety and depression symptoms increases the child’s chances of developing various emotional, behavioural, and cognitive issues later in life. These may include depression, anxiety, Attention Deficit Hyperactivity Disorder (ADHD), and/or conduct disorders. There is an increased risk for other outcomes as well, including a reduction in telomere length, which may be indicative of an accelerated life history (13).

The Edinburgh Postnatal Depression Scale (EPDS) is the most widely used tool to detect perinatal depression, as “its accuracy and psychometric properties are the most established of any depression screening tool in a range of perinatal populations” (14, 15). It is an agile ten-item scale that women can complete in 2 or 3 min in the waiting room, online, or with a professional. It has been observed that its detection capacity is even higher than that of a clinical interview because when interviewed by a professional, new mothers may not admit to having depressive symptoms so as not to feel an overwhelming sense of shame and guilt for being “worse mothers” than they think they should be (16).

Different international studies have validated a single instrument to systematise detecting depressive and anxious symptoms in pregnancy and postpartum. They found a three-factor structural solution for the Edinburgh Postnatal Depression Scale (EPDS): Depressive symptoms (Items 7, 8, 9, and 10), Anhedonia (Items 1 and 2) and Anxiety symptoms (Items 3, 4, and 5) (17–19). Studies in Australia, France and Denmark have identified an Anxiety subscale called EPDS-3A, composed of Items 3, 4, and 5, as a valid and efficient measure of perinatal anxiety (20, 21). This subscale can detect at-risk women who are not detected with routine screening using the EPDS (22), being item 10 particularly relevant for the exploration of suicidal ideation (23). Other studies have found that the anxiety factor in the EPDS is robust and correlates with other measures of anxiety (18, 24). Some authors suggest that all three items can be used for anxiety screening through a cut-off point of 6 or more (25).

The first study in Spanish population to analyse the factorial structure of the EPDS was carried out in 2018. Although the authors found a three-factor solution (depression, anxiety and anhedonia), they did not assess the predictive validity of the anxiety factor (26). In this factorial structure, the Anxiety subscale of the Spanish version consisted of 4 items, including Item 6 (“things are getting on top of me” translated in the Spanish version as “las cosas me oprimen o me agobian”), as previously underlined in the Hispanic population in USA and Mexico (27–29). To date, no validation of the assessment of perinatal anxiety using the EPDS-A subscale has been carried out in Spanish population.

A single pre- and post-natal instrument is useful because it allows longitudinal and predictive studies, correlating with risk and protective factors and examining the evolution of depressive-anxious symptoms and their comorbidity (6, 7). In many countries, as in Spain, a screening protocol for depressive and anxious symptomatology, the basis for preventive intervention recommended by the World Health Organisation (WHO), has not yet been established (30). Perinatal prevention has the highest rate of return on investment in human capital when applying antenatal and postnatal programs (31).

Given that there is currently no updated protocol or clinical practice guideline for the Spanish territory (32), the present study aims to evaluate in Spanish population the validity of the EPDS Anxiety subscale (EPDS-A) for the detection of anxiety and depression simultaneously with a single instrument in pregnancy and postpartum. For this purpose, we first aim to confirm the three-factor structure of the Spanish EPDS. Next, the instrument’s psychometric properties and internal consistency are analysed Then, its invariance in pregnancy and postpartum is tested, and finally, the predictive validity of the EPDS-A is evaluated. As a secondary objective, risk factors associated with depressive-anxious symptomatology are explored.

## 2 Methods

### 2.1 Design

This is a non-probabilistic cross-sectional correlational study, carried out with the entire population of the Roquetes-Canteres health center that met the inclusion criteria and consented to participate in the study.

### 2.2 Participants

Participants were 161 women aged between 18 and 46 years. Eighty were evaluated during pregnancy, and 81 within six months postpartum. Participants were recruited from a Primary Healthcare Center in Barcelona, Spain. The sample comprises all the women evaluated at the centre who met the inclusion and exclusion criteria and agreed to participate in the study.

#### 2.2.1 Inclusion criteria

pregnant women from the 12th week of pregnancy or women in the postpartum period up to 6 months postpartum.

#### 2.2.2 Exclusion criteria

Individuals were excluded from the study if they were under 18 years of age, not assigned to the provider centre, unable to communicate in Spanish, or presented intellectual disability or psychiatric pathology of sufficient severity to require immediate intensive treatment. Failure to answer any of the questionnaires was also a reason for excluding the participant from the sample.

Considering these criteria, 73 of the 234 participants initially evaluated were excluded from the study.

**Figure 1:**
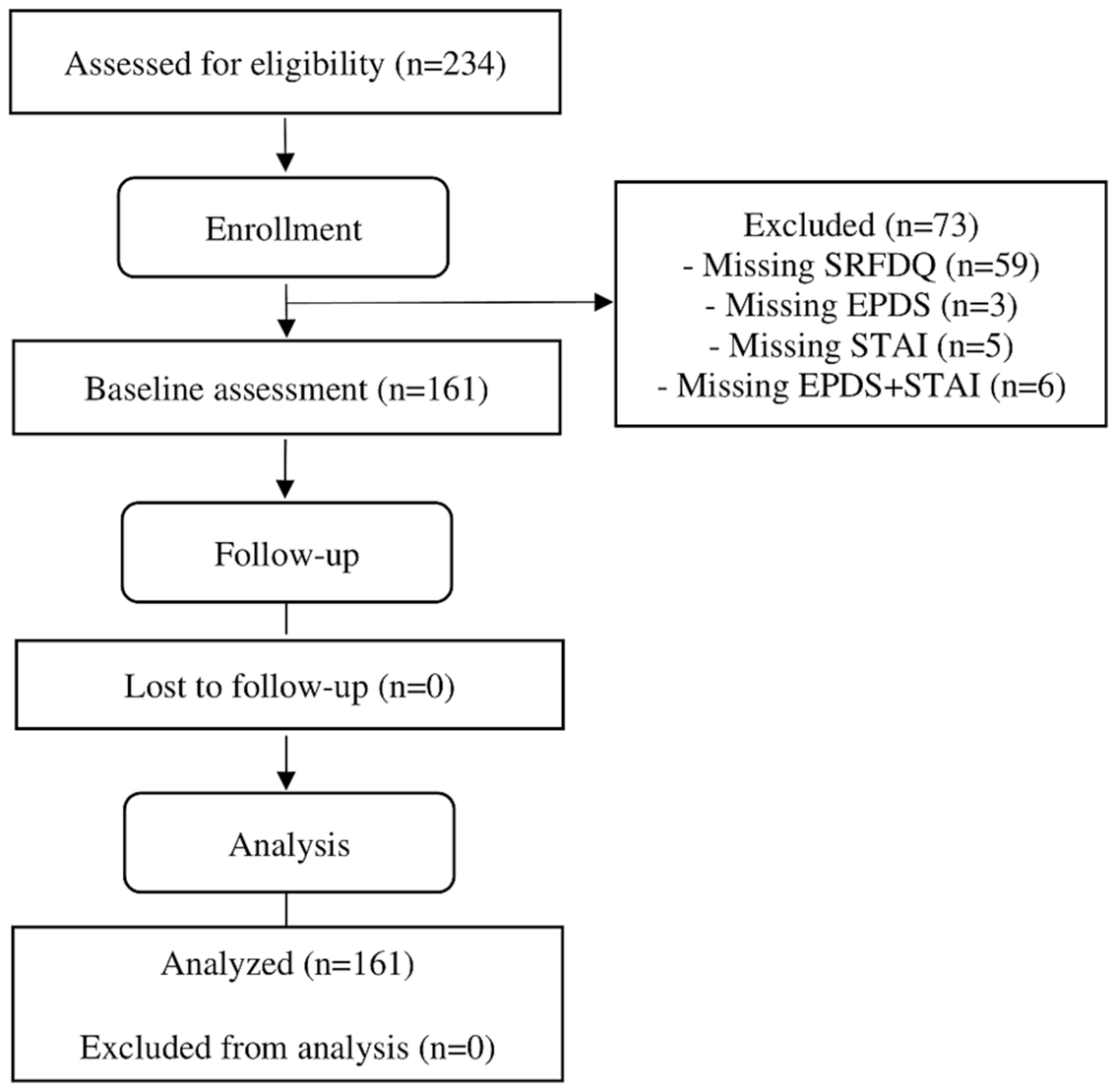
Flowchart of participants (modified for non-randomised trial design).

Figure 1 presents the flowchart of Participants.

Table 1 shows the characteristics of the sample. The significant representation of migrant women whose countries of origin are outside the EU is noteworthy (50% of pregnant women, 40.5% of postpartum women). A large number of pregnant women were unemployed (33.8%), with an even higher percentage of unemployed women in the postpartum group (43.2%). About 40% of pregnancies in both groups were unplanned. Also noteworthy was the high percentage of women who had suffered a previous miscarriage (47.6% of the pregnant women and 32.1% of the postpartum group). Among the adverse experiences suffered recently, stressful events in the last 24 months and the loss of important family members stand out, between 35% and 40% in both groups.

**Table 1:** Sample characterisation.

	Pregnant women *N =* 80 (%)	Postpartum *N =* 81 (%)
Age *M* (SD)	29.74 (6.22)	29.77 (5.33)
Pregnancy week/Postpartum week *M* (SD)	18.27 (7.7)	6.12 (6.4)
Living alone	6 (7.5)	4 (4.9)
Living in one room	10 (12.5)	15 (18.5)
Origin outside Europe	40 (50)	49 (40.5)
Basic education	19 (23.8)	13 (16.0)
Unemployed	27 (33.8)	35 (43.2)
First pregnancy/child	39 (48.8.)	23 (28.4)
Previous miscarriage	38 (47.6)	26 (32.1)
Spontaneous miscarriage	24 (30.0)	16 (19.8)
Unplanned pregnancy	32 (40.0)	34 (42.0)
Unwanted pregnancy	2 (2.5)	6 (7.4)
Lack of partner support	9 (11.3)	8 (9.9)
Lack of family support	9 (11.3)	15 (18.8)
Lack of social relationships	3 (3.8)	11 (13.6)
Hospitalisation	0 (0)	7 (8.6)
History of physical health problems	8 (10.0)	5 (6.2)
History of mental health problems	22 (27.5)	8 (9.9)
Consumption of psychotropic drugs	19 (23.8)	5 (6.2)
Consumption of alcohol	1 (1.3)	1 (1.2)
Consumption of tobacco	12 (15.0)	13 (16.0)
Stressful events in the last 24 months	31 (38.8)	32 (39.5)
Previous experience of violence	11 (13.8)	4 (4.9)
Experience of mistreatment in infancy	7 (8.8)	5 (6.2)
Important family member losses	28 (35.0)	31 (38.3)
Recent severe illness of a loved one	18 (22.5)	17 (21.0)

### 2.3 Measures

The Sociodemographic and Risk Factors Data Questionnaire (SRFDQ) is a questionnaire created *ad hoc* by the research team that collects data on the following variables: sociodemographics, previous indicators, psychological data, mental health history and significant family losses (33).

The Edinburgh Postnatal Depression Scale (EPDS) (14), although initially validated to assess depressive symptomatology in the postnatal period, was later validated in Spanish pregnant women (34, 35). It is a self-applied scale with 10 Likert-type items. It has shown adequate psychometric properties in several languages, cultures and samples (15, 16). A score equal to or greater than 9 is considered an indication of risk.

The State–Trait Anxiety Inventory (STAI) is one of the most widely used instruments for measuring anxiety. It distinguishes state and trait anxiety and has proven to be a valid and sensitive instrument for measuring anxiety in different populations, including perinatal population (36, 37).

### 2.4 Procedure

A multidisciplinary team (midwife, doctor, nurse, social worker, paediatrician and psychologist) administered these instruments and monitored the results. After an introductory visit, the primary care provider who had contact with the women explained the study, collected informed consent and administered the SRFDQ. Subsequently, the midwife (early in pregnancy) and/or nurse or physician (early-6-month postpartum) administered the Depression (EPDS) and Anxiety (STAI) questionnaires.

A perinatal psychologist contacted all the women with scores above the clinical cut-off point in the EPDS and the STAI to offer manualised, preventive psychological treatment based on scientific evidence (37, 38).

Before data collection, the entire Primary Care team was trained through workshops and clinical sessions in administering the three instruments. Throughout the study, continuous team training was maintained with three annual clinical sessions by the research team.

This research was approved by the Research Ethics Committee of the FPCEE Blanquerna Universitat Ramon Llull, code number 2122019D. The research procedures of the prospective study were approved by the IDIAP Research Ethics Committee Jordi Gol i Gurina, code number P715.

### 2.5 Statistical analysis

In accord with the study’s main objective, a one-dimensional model consisting of the 10 items of the EPDS (M1) was compared with the three-dimensional model proposed in the Spanish version of the EPDS (26).

To confirm which model best fit the data, confirmatory factor analysis (CFA) of each model was carried out. For this purpose, we used the AMOS 25.0 program with the maximum likelihood (ML) method (39). We evaluated the goodness of fit of the data to the proposed measurement models through the chi-square (χ^2^) test of the equality contrast matrix, which should be nonsignificant or present low values, and the ratio between chi-square and the degrees of freedom of the model (χ^2^/*df*), which should be less than 3 (40, 41). We also used the root mean square error of approximation (RMSEA) and its 90% confidence interval (CI), considering values between 0.05 to 0.08 acceptable, and values less than 0.05 very good. Likewise, we also used the goodness of fit index (GFI), the comparative fit index (CFI), whose values should be greater than 0.90 (42), and the standardised root mean residual (SRMR), whose value must be less than 0.08 to be considered acceptable (43).

Once we had confirmed which model best fit the data, we analysed its psychometric properties. For this purpose, we analysed the distribution of the EPDS items. To identify items that were not discriminatory, we used the following criteria: (a) mean plus/minus one standard deviation of the subscale’s mean; (b) standard deviation less than 0.5; (c) correlation values lower than 0.40 with the corresponding dimension; and (d) increase in the total reliability of the subscale by more than 0.3 points when eliminating the item (44, 45). Finally, we calculated the structural coefficients between each item and the overall score of their corresponding dimension.

Next, we analysed the internal consistency of the three dimensions that make up the EPDS through Cronbach’s alpha (α) reliability coefficient and McDonald’s omega (ω) reliability, a more accurate estimator than alpha in the absence of tau-equivalence or the presence of asymmetric items (46, 47).

The next step was to analyse the invariance of the EPDS during pregnancy and postpartum. For this purpose, we performed multigroup confirmatory factor analysis (AFCMG) with the AMOS 25.0 program. This analysis is carried out from a succession of hierarchically nested models. Every model was tested using maximum likelihood and based on a covariance matrix (48). All models were tested according to the configural model (M1). The first model (M1) is the base model and allows analysing the configural invariance. In this model, no equality restrictions are established in any of the model’s parameters. The next models establish equality constraints between the two groups for different parameters. In the second model, we ensured that the factor loadings were equivalent (M2). Next, we tested that the structural composition of the loads was the same (M3). Following this, the same structure of variance and covariance was fixed between factors (M4). Then, the residual structure was fixed for the two groups (M5). Finally, the same loadings for all the measurement errors were constrained across samples (M6). We used the value of ΔCFI and not the differences in chi-square because a robust estimation method must be used to interpret the results. Specifically, values lower than 0.01 of this indicator were a criterion to determine the invariance between models (49).

Next, to evaluate the predictive validity of the EPDS-A, we analysed the association between the Anxiety subscale of the EPDS (EPDS-A) and the State and Trait subscales of the STAI in Spanish population.

Lastly, to determine the cut-off value of the EPDS-A score that provides the best values for the sensitivity and specificity indicators, we used the Receiver Operating Characteristics (ROC) curve analysis (50, 51). We calculated the area under the curve (AUC), its 95% CI, and the asymptotic significance value, presenting the ROC curve’s characteristic graphic representation. This methodology allows selecting cut-off points that are better suited to the objective of our study, considering that the reduction or increase in sensitivity implies an increase or reduction of specificity, and vice versa.

Finally, in line with the secondary objective of the study, we established two groups: a risk and a non-risk group according to EPDS and STAI-S and STAI-T scores. Differentiation into two groups allowed a new analysis of risk factors associated with prenatal mental health to be carried out. The risk group comprised pregnant women who obtained high scores in at least two of the three instruments. We applied the chi-square test to study the relationship between the associated factors and their odds ratio. We also used Mann–Whitney’s μ to compare the means of the risk and non-risk groups in the three EPDS subscales, the STAI-S and the STAI-T.

## 3 Results

### 3.1 Validity of the EPDS anxiety subscale (EPDS-A)

The EFA-driven three-factor structure of the EPDS (M2), consisting of an Anhedonia factor (Items 1, 2 and 10), an Anxiety factor (Items 3, 4, 5 and 6) and a Depression factor (Items 7, 8, and 9), was the best measurement model for the current data compared to the alternative model tested, χ^2^ = 34.592, *df* = 32, *p* = 0.34; χ^2^/*df* = 1.08; RMSEA = 0.023, 90% CI [0.000, 0.064], CFI = 0.996, GFI = 0.960 (See Table 2).

**Table 2:** Confirmatory factor analyses of the EPDS.

Model	χ^2^	df	χ^2^/df	*p*	RMSEA	GFI	CFI	SRMR
M1	93.190	35	2.66	0.000	0.102	0.885	0.906	0.065
M2	34.592	32	1.08	0.345	0.023	0.960	0.996	0.044

**Figure 2:**
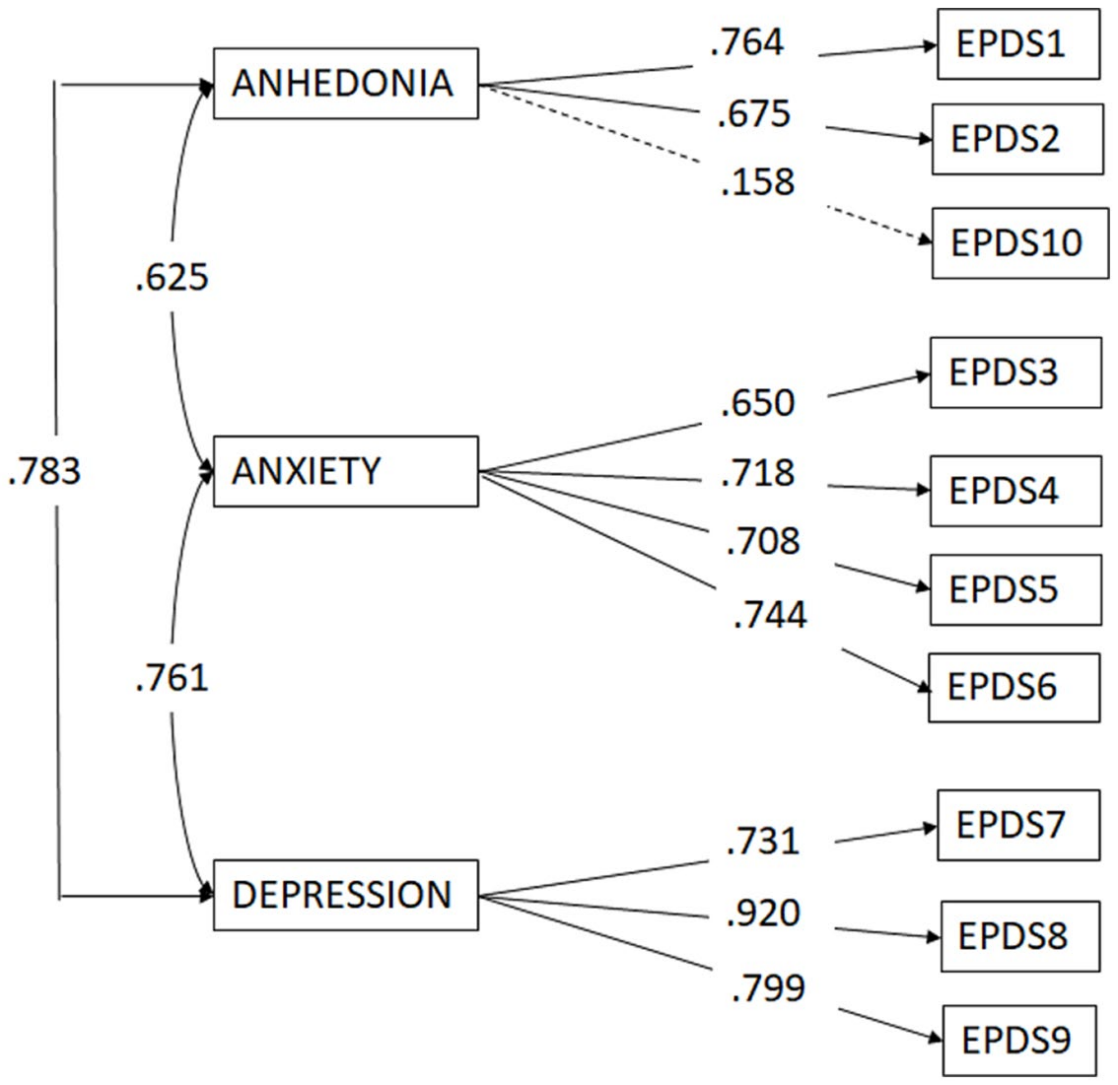
Confirmatory factor analysis of the EPDS.

As shown in Figure 2, the structural coefficients of all the items with their corresponding factor are statistically significant, with values above 0.60, except for Item 10 on the Anhedonia factor, which was nonsignificant.

Table 3 presents the descriptive and reliability data of the EPDS subscales. The Anhedonia subscale showed lower reliability values compared to the Anxiety and Depression subscales.

**Table 3:** Descriptive and reliability data of the three subscales of the EPDS.

Dimension	Range	*M*	SD	SK	Ku	*α*	*ω*
Anhedonia	0–9	0.776	1.167	1.783	2.933	0.521	0.686
Anxiety	0–12	4.845	3.018	0.051	−0.981	0.797	0.799
Depression	0–9	3.255	1.953	0.686	0.383	0.855	0.859

SK, skewness; Ku, kurtosis.

Table 4 shows the descriptive and discrimination data of the items that make up each of the subscales of the EPDS. The internal consistency of the Anxiety and Depression dimensions was adequate, in both cases around 0.80. However, although reaching acceptable values, the internal consistency index of the Anhedonia dimension was low. This is due to the functioning of Item 10, which is the only one that violates the item retention criteria mentioned in the previous section. On the one hand, the standard deviation was less than 0.5, and the correlation value with the corresponding subscale was 0.009, well below 0.40. On the other hand, if we eliminated that item, the reliability of the subscale would rise from 0.521 to 0.682, whereas it would decrease drastically if we eliminated either of the other two items that make up the Anhedonia dimension. The extremely high skewness and kurtosis indices indicate a very homogeneous distribution of the responses to Item 10, accumulating in the lowest values.

**Table 4:** Descriptive and discrimination data of the EPDS items.

Dimension	Item	Range	*M*	SD	SK	Ku	*r*	*α*-item
Anhedonia	1	0–3	0.30	0.581	1.967	3.712	0.489	0.127
	2	0–3	0.43	0.677	1.790	3.534	0.493	0.103
	10	0–3	0.04	0.303	7.973	68.272	0.009	0.682
Anxiety	3	0–3	1.26	0.952	−0.061	−1.188	0.548	0.776
	4	0–3	1.26	0.972	−0.132	−1.313	0.667	0.718
	5	0–3	0.94	0.940	0.399	−1.198	0.608	0.748
	6	0–3	1.39	0.962	−0.116	−1.045	0.615	0.744
Depression	7	0–3	0.48	0.791	1.529	1.349	0.671	0.849
	8	0–3	0.53	0.783	1.448	1.469	0.791	0.734
	9	0–3	0.58	0.755	1.319	1.506	0.722	0.802

SK, skewness; Ku, kurtosis; *α*-item, alpha value if item is removed.

Next, we tested the invariance of the three-dimensional model of the EPDS between pregnant and postpartum women. Table 5 shows that the base model (M1) had acceptable fit indices, so the configural invariance between pregnancy and postpartum could be assumed. We observed that the decrease in CFI between M1 and M2 (ΔCFI = 0.007) was less than 0.01, so the criterion of metric invariance could be assumed in both groups (pregnancy and postpartum). Between M2 and M3, the CFI decline was less than 0.01 (ΔCFI = −0.002). This means that the factor loadings did not differ in the structure models across the two groups. In Model 4 (M4), the variances and covariances of the three dimensions of the EPDS were fixed. In this model, the differences in CFI were also less than 0.01 (ΔCFI = 0.001), indicating that the dimensions had a similar meaning for the pregnancy and postpartum groups and followed the same relational pattern. In Model 5 (M5) and Model 6 (M6), the structural residual invariance and the residual measurement were also fixed. The decrease in CFI between M4 and M5 allowed the structural residual invariance to be maintained (ΔCFI = 0.004). However, the difference between models M5 and M6 (ΔCFI = 0.015) did not guarantee the structural residual measurement.

**Table 5:** Global fit indices for the invariance of the EPDS pre and postpartum.

	*χ* ^2^	df	*p*	*χ*^2^/df	CFI	TLI	SRMR	RMSEA	ΔCFI
M1. Configural model	65,629	64	0.420	1.03	0.997	0.996	0.0507	0.013	-
M2. Measurement weights invariance	77,192	71	0.287	1.09	0.990	0.987	0.0539	0.023	0.007
M3. Structrural weights invariance	77,624	73	0.334	1.06	0.992	0.991	0.0532	0.020	−0.002
M4. Structural covariances invariance	79,395	74	0.313	1.07	0.991	0.989	0.0606	0.021	0.001
M5. Structural residuals invariance	79,853	77	0.389	1.04	0.995	0.995	0.0603	0.015	0.004
M6. Measurement residuals invariance	99,325	87	0.173	1.14	0.980	0.979	0.0564	0.030	0.015

*χ*^2^, chi-square; Df, degree of freedom; CFI, comparative fit index; TLI, Tucker Lewis coefficient; SRMR, standardised root mean square residual; RMSEA: root mean square error of approximation.

The next step was to evaluate the predictive validity of the EPDS-A. The associations between the Anxiety subscale of the EPDS (EPDS-A) and the State and Trait dimensions of the STAI were significant both in pregnancy and postpartum. Specifically, we observed that the EPDS-A was significantly and positively associated with the STAI-State dimension during pregnancy (*r* = 0.64, *p* < 0.001) and postpartum (*r* = 0.72, *p* < 0.001). The EPDS-A was also significantly and positively associated with the STAI-Trait dimension during pregnancy (*r* = 0.71, *p* < 0.001) and postpartum (*r* = 0.73, *p* < 0.001). These associations were high (see Table 6).

**Table 6:** Correlations between the EPDS-A and the STAI during pre and postpartum.

	EPDS-A	
	Pregnancy	Postpartum
STAI-state	0.64^**^	0.72^**^
STAI-trait	0.71^**^	0.73^**^

*p < 0.05. **p < 0.001.

**Figure 3:**
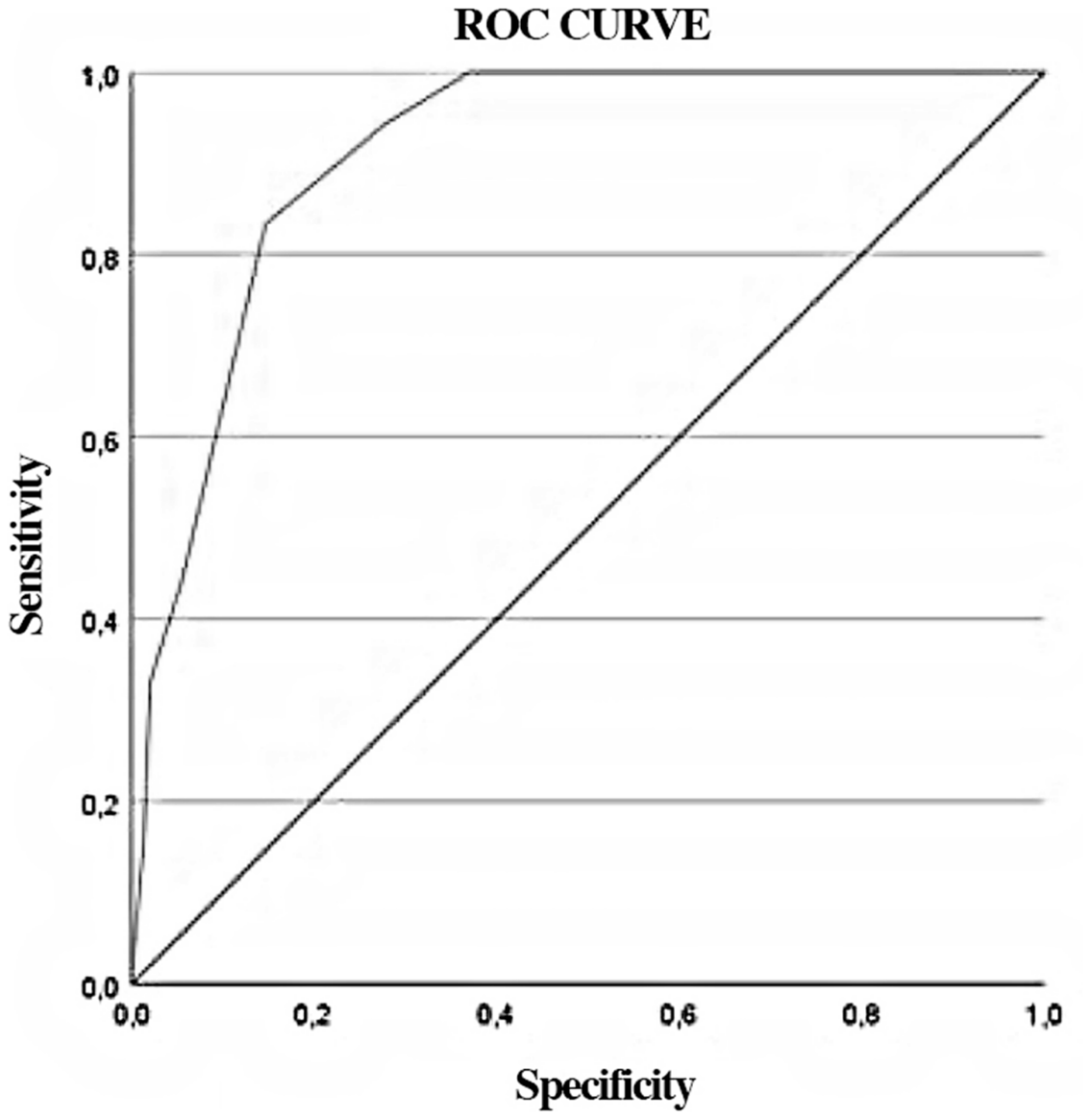
ROC curve for EPDS-A with STAI scores as the standard (PD ≥ 40).

Finally, Figure 3 presents the ROC curve obtained for the EPDS-A dimension, showing its ability to discriminate between women who present anxiety scores at the clinical level and those who do not. The diagonal of the figure represents the condition of null discrimination, and any curve that moves away from this diagonal and covers a greater area towards the upper left corner indicates a greater diagnostic utility.

Table 7 presents the values obtained for the area under the curve (AUC), sensitivity, specificity, and cut-off point, considering the criterion of prioritisation of sensitivity because this instrument is designed to screen for anxiety. The cut-off point for this instrument is 3.5, but as the scores obtained are whole numbers, a score of 4 points will be considered as the cut-off point.

**Table 7:** Significance of AUC for the EPDS-A, sensitivity, specificity, and cut-off point.

	AUC	95% confidence interval	Standard error	*p*	Sensitivity	Specificity	Cut-off point
EPDS-A	0.91	0.859, 0.962	0.026	0.000	1.000	0.594	3.5

### 3.2 Risk factors associated with the mental health of women in the perinatal stage

Of the 161 women who agreed to participate in the study, 56 women (34.8%) presented an EPDS score indicating a risk of depression. On the STAI, 92 women (57.1%) had clinically significant scores for State anxiety and 76 women (47.2%) had significant scores for Trait anxiety.

Considering the individuals who met the criteria for risk of depressive and anxious symptomatology, we obtained a total sample of 70 patients, representing a rate of 43.5%.

We obtained statistically significant differences in the Mann–Whitney’s μ when comparing the means of the risk and non-risk groups in the three EPDS subscales: Anhedonia (*p* < 0.001; risk group *M* = 1.50, SD *=* 1.40, vs. non-risk group *M* = 0.22, SD *=* 0.44); Depression (*p* < 0.001; risk group *M* = 4.40, SD *=* 1.91, vs. non-risk group *M* = 2.37, SD *=* 1.48); and Anxiety (*p* < 0.001; risk group *M* = 7.14, SD *=* 2.21, vs. non-risk group *M* = 3.08, SD *=* 2.27). We also found differences when comparing the scores of the STAI (State: *p* < 0.001 risk group *M* = 26.35, SD *=* 9.68, vs. non-risk group *M* = 9.53, SD *=* 5.84; and Trait: *p* < 0.001 risk group *M* = 28.42, SD *=* 8.41, vs. non-risk group *M* = 11.88, SD *=* 5.70).

The chi-square statistic was employed to compare the risk and non-risk groups and identify the statistically significant associated factors.

Table 8 shows the characteristics of the female population with risk and non-risk scores, indicating statistical significance.

**Table 8:** Sociodemographic description and associated factors for the non-risk and risk groups.

	Non-risk group *N =* 91 (%)	Risk group *N =* 70 (%)	*p*-value	Odds ratio
Age *M* (SD)	29.79 (5.41)	29.70 (6.25)	0.764	
Living alone	6 (6.6)	4 (5.7)	0.052	
Living in one room	10 (11.0)	15 (21.4)	0.070	
Origin outside Europe	48 (52.7)	41 (58.6)	0.461	
Basic education	58 (63.7)	40 (57.1)	0.395	
Unemployed*	26 (28.6)	36 (51.4)	0.003	2.647
First pregnancy/child*	59 (64.8)	34 (48.6)	0.038	0.512
Previous miscarriage	33 (36.3)	31 (44.3)	0.303	
Spontaneous miscarriages	23 (25.3)	16 (22.9)	0.723	
Unplanned pregnancy	34 (37.4)	31 (45.7)	0.285	
Unwanted pregnancy	2 (2.2)	6 (8.6)	0.065	
Lack of partner support*	4 (4.4)	13 (18.6)	0.004	4.961
Lack of family support*	7 (7.7)	17 (24.3)	0.003	3.849
Lack of social relationships	5 (5.5)	9 (12.9)	0.100	
Hospitalisation*	1 (1.1)	6 (8.6)	0.022	8.340
History of physical health problems	8 (8.9)	5 (7.1)	0.688	
History of mental health problems*	12 (13.2)	18 (25.7)	0.043	2.279
Consumption of alcohol	0 (0)	2 (2.9)	0.105	
Consumption of tobacco	14 (15.4)	11 (15.7)	0.954	
Stressful events in the last 24 months*	27 (29.7)	36 (51.4)	0.005	2.509
Previous experience of violence	6 (6.6)	9 (12.9)	0.175	
Experience of mistreatment in infancy*	3 (3.3)	9 (12.9)	0.022	4.328
Important family member losses	30 (33.0)	29 (41.4)	0.269	
Recent severe illness of a loved one	20 (22.0)	15 (21.4)	0.933	

*p < 0.05. **p < 0.001.

## 4 Discussion

The results confirmed the three-factor structure described above by the Spanish study (26) and the invariance of the measurement model between pregnancy and postpartum. We confirmed the structure found in the aforementioned study, finding a three-factor solution that we called Depressive symptoms (Items 7, 8, and 9), Anhedonia (Items 1, 2, and 10) and Anxiety symptoms (Items 3, 4, 5, and 6). The present study analysed the invariance of the measurement model between a group of pregnant women and a group of women in the postpartum period, finding that the trifactorial structure remains invariant. To our knowledge, this analysis has not been done before.

The factor analysis of the EPDS items performed in various samples in different countries has shown several factorial solutions (21, 52). Some studies have obtained a three-factor solution (20, 53, 54). The results of these studies indicate the existence of an Anxiety subscale within the EPDS and, therefore, the possibility of using the EPDS as a screening instrument both for depression and anxiety in the perinatal period.

However, numerous studies have found that this subscale comprises three items (3, 4, and 5) (21, 23, 55). In contrast, in the present study, the Anxiety subscale has four items (3, 4, 5, and 6), as in the first study that analysed the EPDS structure in a Spanish sample (26). We propose that the Spanish translation of the EPDS can help explain the difference in Item 6 (“Las cosas me oprimen o agobian” – “Things have been getting on top of me”), being part of the Anxiety subscale instead of the Depression subscale, according to previous studies (27–29).

The contribution of Item 10 (“He pensado en hacerme daño a mí misma” – “The thought of harming myself has occurred to me”) requires some reflection. It has been included within the Anhedonia factor, following the structure of the reference study (26). The internal consistency of the instrument and the three subscales was adequate, as were the adequacy indices of the items that compose it, except for Item 10. The results suggest that the functioning of Item 10 differs significantly from the rest of the items that make up the EPDS. The analysis of the distribution of the responses to Item 10 indicates that a positive response to this item is very infrequent, even when the rest of the scale’s items obtain high scores. We consider that this is due to the differential content of the item, as it is the only item that refers to suicidal ideation.

On the other hand, although the three-factor solution includes Item 10 in the Anhedonia factor, fitting the data adequately, the structural coefficient of this item was not significant. In other studies, it has been grouped differently and even formed a factor independently (21, 23, 56). Therefore, it is necessary to continue studying the functioning of Item 10 and assess its validity for detecting self-injury risk in the perinatal period. Some authors advocate eliminating Item 10 from the EPDS and assessing suicidal ideation through other tools (57). As many health professionals have difficulties exploring suicidal ideation, this decision could constitute an obstacle to its detection. Alternatively, we consider that the positive response to this item is an alarm signal that requires a subsequent clinical interview, as other authors maintain (25).

Some studies have found that the EPDS Anxiety subscale or factor is robust and correlates with other measures of anxiety both in pregnancy and postpartum (19, 23). In this study, we analysed the EPDS-A with the State/Trait STAI, finding moderate to high correlations in all cases, although slightly higher in the postpartum period.

Based on these results, we can conclude that the Anxiety subscale (EPDS-A) of the Spanish EPDS, composed of Items 3, 4, 5 and 6, is a reliable and valid measure for the screening of anxiety in women during pregnancy and postpartum. The area under the ROC curve, with a value of 0.91, indicates that the score on the Anxiety subscale of the EPDS shows good diagnostic value for anxious symptomatology in the perinatal period. It is worth mentioning that the sensitivity of the EPDS-A was prioritised because it is a screening instrument. The high sensitivity and low specificity of the EPDS-A means that this subscale will yield few false negatives and many false positives. As a screening test, this tendency would, on the other hand, prevent women with anxious symptomatology during this period from not being identified and further evaluated, and treated if necessary. The cut-off point was 4 or higher, which coincides with another study in Australian population (58). However, other studies found an Anxiety subscale composed of three items with higher cut-off points, so their results are not comparable. Therefore, it is important to incorporate a clinical sample to corroborate the present study.

Concerning the risk factors studied in the previous study (33), two factors are added: being a new mother and having been hospitalised in the perinatal period. The following are confirmed as significant factors: unemployment, lack of partner support, lack of family support, previous miscarriage, history of mental health problems, stressful events in the last 24 months, and experience of mistreatment in infancy. The importance of risk factors in perinatal mental health has been previously studied, and they are particularly relevant for bio-psycho-social preventive interventions, expanding the complexity of our comprehension and intervention in maternal and child health, which is highly cost-effective (31).

In terms of their applicability and implications, we need questionnaires that enable the efficient and straightforward screening of the most common mental health symptoms in the population (13). In Spain, there is a need for a unified screening protocol that not only facilitates the detection but also the implementation of preventive interventions to mitigate the well-studied and well-known risk associated with perinatal depression and anxiety. This applies to both the mother’s well-being, the infant’s development, and the quality of the relationship being established between them.

### 4.1 Limitations

This study is not without its limitations. First, it is a non-probabilistic cross-sectional design that limits the generalizability of the results and the analysis of the relationships between variables. In addition, the sample belongs to a population that is geographically localised, although the high representation of migrants has already been mentioned. In future studies, we should look for larger and more heterogeneous samples that represent different populations, as well as choose longitudinal designs to explore in depth the causal relationships between variables, as well as to extend the validity study of the EPDS-A. On the other hand, only self-report instruments have been used as a source of information. The data could be enriched, for example, through interviews or interaction observation.

### 4.2 Strengths

This study has made it possible to analyse the applicability of the EPDS in pregnancy and postpartum assessment. In addition, using complex data analysis techniques, it has corroborated the reliability and construct validity of the instrument, as well as the convergent validity of the Anxiety subscale and data on its specificity and sensitivity. Thus, it supports using the EPDS for the simultaneous assessment of depressive and anxious symptoms in pregnant and postpartum women through an instrument that is simple and quick to apply.

## 5 Conclusion

We consider the EPDS an agile tool (16) for the early and simultaneous detection of depressive and anxious symptoms both in pregnancy and postpartum. Therefore, it could be included in clinical practice guidelines for Spanish population to advance towards a common basis for early detection and preventive interventions (30).

## Data availability statement

The raw data supporting the conclusions of this article will be made available by the authors, without undue reservation.

## Ethics statement

The studies involving humans were approved by IDIAP Jordi Gol Foundation P15/038 and FPCEE Blanquerna Universitat Ramon Llull; code number 2122019D. The studies were conducted in accordance with the local legislation and institutional requirements. The participants provided their written informed consent to participate in this study.

## Author contributions

MG: Conceptualization, Data curation, Funding acquisition, Investigation, Resources, Validation, Writing – original draft, Writing – review & editing. LG: Data curation, Formal analysis, Funding acquisition, Investigation, Methodology, Validation, Writing – original draft, Writing – review & editing. EC: Data curation, Formal analysis, Investigation, Methodology, Resources, Software, Writing – original draft, Writing – review & editing. LI: Data curation, Formal analysis, Funding acquisition, Investigation, Methodology, Project administration, Resources, Software, Validation, Writing – original draft, Writing – review & editing. EP: Data curation, Investigation, Project administration, Resources, Validation, Writing – review & editing. JF: Investigation, Project administration, Resources, Supervision, Visualization, Writing – review & editing. CS: Data curation, Investigation, Project administration, Resources, Writing – review & editing. AL: Conceptualization, Formal analysis, Funding acquisition, Investigation, Resources, Supervision, Validation, Visualization, Writing – original draft, Writing – review & editing. EA-P: Formal analysis, Funding acquisition, Investigation, Methodology, Project administration, Resources, Supervision, Validation, Visualization, Writing – original draft, Writing – review & editing, Conceptualization.
